# Response to Letter to the Editor: Prospective Validation of PaDd—A Roadmap

**DOI:** 10.5041/RMMJ.10569

**Published:** 2026-01-28

**Authors:** Ramon Cohen, Daniel Elbirt

**Affiliations:** 1 Faculty of Medicine, Hebrew University of Jerusalem, Jerusalem, Israel; 2 Department of Internal Medicine B, Kaplan Medical Center, Rehovot, Israel; 3 Department of Clinical Immunology Allergy and AIDS, Kaplan Medical Center, Rehovot, Israel

**Keywords:** Elderly, PaDd, Padua

## To the Editor

We thank Dr Vijayasimha and colleagues for their thoughtful analysis of our work and their proposal for prospective validation of the Padua × D-dimer (PaDd) score.^1^ Their roadmap provides a rigorous framework that addresses critical gaps in our single-center retrospective design. We welcome this opportunity to clarify key methodological considerations and invite the global research community to collaborate on next-generation studies.

Our retrospective cohort demonstrated that PaDd improves specificity (9% to 32%) while maintaining 100% sensitivity for pulmonary embolism (PE) exclusion in adults ≥65 years.^2^ However, we recognize that retrospective findings—particularly those derived from hospitalized patients—require rigorous prospective validation before clinical implementation. Dr Vijayasimha correctly identifies four domains where our preliminary data must be stress-tested: comparator-anchored validation—need to compare against validate score like age-adjusted D-dimer (AADD), assay-specific calibration, system-level equity, and subsegmental PE management. We added four other important domains to take into consideration for prospective study.

We address each domain below, adding nuances from our dataset that prospective investigators should consider.

### (1) Selection bias and care pathway stratification

Our cohort included only patients admitted to internal medicine wards; outpatients discharged were excluded. This design likely enriched our sample for sicker individuals—regardless of PE status—potentially inflating baseline Padua scores. In prospective studies, we recommend sub-analysis with stratifying enrollment by care pathway (emergency department discharge or admission) and reporting PaDd performance separately for each stratum. This approach will clarify whether PaDd generalizes to lower-acuity settings where diagnostic uncertainty is highest.

### (2) Operationalizing “reduced mobility” in the Padua score

The Padua Prediction Score assigns 3 points for “reduced mobility ≥3 days,” yet this term lacks standardized definition.^3^ In our study, mobility assessment relied on retrospective chart review, introducing potential inter-rater variability. Prospective trials must operationalize this criterion using validated instruments. We propose pilot-testing multiple templates (for example):

- Template A (Quantitative): Mobility reduction of 25%, 50%, 75%, or bedbound relative to baseline.
- Template B (Functional): Three-day recall: “usual activity,” “mostly chair-bound,” “mostly bedbound.”
- Template C (Objective): Step count via wearable devices (if feasible).

We propose five pilot-testing templates (see supplement (. A nested substudy comparing these templates against actigraphy-measured step counts would identify which definition best discriminates PE risk while maintaining inter-rater reliability and feasibility in real-world settings.

### (3) NOAC exposure and the PaDd/Activated Partial Thromboplastin Time (aPTT) ratio

Non-vitamin K antagonist oral anticoagulants (NOACs) prolong aPTT and theoretically reduce PE incidence.^4^ In our cohort, 7% of PE-negative and 3% of PE-positive patients were on NOACs or low-molecular-weight heparin. While we did not exclude these patients, their inclusion may have biased the PaDd/aPTT ratio downward. Prospective studies should either exclude NOAC-treated patients or conduct sensitivity analyses stratified by anticoagulation status. Additionally, investigators should collect data on NOAC timing relative to blood sampling, as recent dosing may disproportionately affect aPTT.

### (4) Assay-specific calibration—A call for pragmatic multicenter design

We utilized a single D-dimer assay platform (Siemens Healthcare Diagnostics Products GmbH, Marburg, Germany; Sysmex Corporation, Kobe, Japan). Dr Vijayasimha rightly emphasizes that assay heterogeneity threatens external validity.^5^ The ADJUST-PE trial revealed significant inter-assay variability when applying age-adjusted D-dimer cutoffs.^6^ We strongly endorse assay-stratified validation, wherein each participating center reports outcomes by reagent platform. If feasible, a split-sample substudy—wherein identical specimens are tested on multiple assays—would provide head-to-head calibration data essential for clinical decision support tools.

### (5) Age threshold reconsideration and Padua score of zero

Our inclusion criterion (age ≥65 years) encompasses heterogeneous populations. Healthy 65-year-olds may achieve a Padua score of 0, theoretically limiting PaDd’s utility. Although no PE cases occurred at Padua=0 in our cohort, this may reflect sample size constraints. We recommend sub-analysis restricting prospective enrollment to ≥70 or ≥75 years, with pre-specified sub-analyses by decade (70–79, 80–89, ≥90). This approach aligns with physiological aging milestones and enriches the cohort for patients most likely to benefit from refined risk stratification.

### (6) Subsegmental PE management—Aligning diagnostic and therapeutic parsimony

We classified subsegmental PE as PE-negative based on emerging evidence that anticoagulation withholding is safe in selected cases.^7^ However, prospective protocols must predefine subsegmental PE management to avoid replacing imaging overuse with treatment overuse. We suggest adopting structured surveillance criteria (e.g. absence of proximal deep vein thrombosis, low bleeding risk, close follow-up access) and tracking 90-day venous thromboembolism and bleeding outcomes. This approach will generate real-world evidence on subsegmental PE natural history in older adults.

### (7) Comparator trials—Benchmarking PaDd against established algorithms

Dr Vijayasimha advocates benchmarking PaDd against AADD, and the YEARS and PEGeD algorithms.^8^,^9^ We agree and further propose comparing PaDd with the Geneva Risk Score, which—unlike Wells criteria—eliminates subjective “PE most likely” judgments.^10^ Moreover we recommend trying other new scores such as D-dimer × Geneva Risk Score. A factorial design testing PaDd ± YEARS versus AADD ± YEARS would isolate PaDd’s incremental value while leveraging YEARS’ proven efficiency.

## CONCLUSION

The letter from Dr Vijayasimha and colleagues exemplifies the collaborative rigor required to translate retrospective signals into practice-changing tools. By addressing selection bias, operationalizing Padua components, controlling for NOAC confounding, and embedding assay calibration, the research community can determine whether PaDd achieves its promise: safer, more equitable PE diagnosis in older adults (more information for validation roadmap in the supplement).

## Supplementary Information



## Abbreviations

NOACs: non-vitamin K antagonist oral anticoagulants
PaDd: D-dimer
PE: pulmonary embolism
