# Supplementary material for: Response to Letter to the Editor: Prospective Validation of PaDd—A Roadmap
Source: Rambam Maimonides Med J. 2026 Jan 28;17(1):e0010. doi: 10.5041/RMMJ.10569 (PMC12857655; doi:10.5041/RMMJ.10569)
Supplement: Supplementary file 1 [file rmmj-17-1-e0010_supplement.docx]

This appendix has been provided by the authors for the benefit of readers

Supplement to Response to Letter to the Editor: Prospective Validation of PaDd—A Roadmap

Cohen R, Elbirt D. Response to Letter to the Editor: Prospective Validation of PaDd—A Roadmap. Rambam Maimonides Med J 2026;17 (1):e0010. doi:10.5041/RMMJ.10569

**In This Supplement**

[Proposed Validation Roadmap 2](#_Toc217297872)

[Operationalizing “Reduced Mobility” in the Padua Score: Evidence-Based Template Proposals 2](#_Toc217297873)

[Background: The Problem with Current Padua Definitions 2](#_Toc217297874)

[Key Problems: 2](#_Toc217297875)

[Proposed Templates for Prospective Validation 2](#_Toc217297876)

[TEMPLATE 1: Percentage-Based Mobility Reduction (Quantitative) 3](#_Toc217297877)

[TEMPLATE 2: Functional Activity Scale (3-Day Recall) 3](#_Toc217297878)

[TEMPLATE 3: Objective Measurement (Wearable Device) 4](#_Toc217297879)

[TEMPLATE 4: Structured Clinical Observation (Nurse-Rated) 4](#_Toc217297880)

[TEMPLATE 5: Hybrid Template (Clinical Judgment + Objective Trigger) 5](#_Toc217297881)

[Recommended Pilot Study Design 5](#_Toc217297882)

[Phase 1: Template Comparison Study (n=200 patients) 5](#_Toc217297883)

[Phase 2: Prospective PaDd Validation (n=1,000 patients) 6](#_Toc217297884)

[Anticipated Results by Template 6](#_Toc217297885)

# Proposed Validation Roadmap

We propose a pragmatic, registry-embedded prospective cohort with the following features:

1. **Multicenter enrollment** (≥5 sites across diverse healthcare systems).
2. **Age ≥65 years**; care pathway stratification (Emergency department [ED]-discharge, ED-admit, direct-admit).
3. **Novel Oral Anticoagulant (NOAC)/heparin sensitivity analysis (or exclusion)**.
4. **Standardized mobility assessment** using pilot-tested templates.
5. **Assay-stratified reporting** with optional split-sample sub-study.
6. **Co-primary endpoints**:
   - Proportion of patients below PaDd threshold (efficiency).
   - 90-day venous thromboembolism incidence in PaDd-negative patients (safety).
7. **Pre-specified sub-analyses**: Age, sex, malignancy, infection, renal function, assay type, anti-antiaggregant treatment, Padua score with several templates of mobility reduction, exclusion of patients less than 70 years old, ED-discharge/admit, by center, D-dimer × Geneva Risk Score, PaDd ± YEARS versus AADD ± YEARS, exclusion if PaDd=0, exclusion if received NOAC/heparin, exclusion if patient has low platelets (need to define).

# Operationalizing “Reduced Mobility” in the Padua Score: Evidence-Based Template Proposals

## Background: The Problem with Current Padua Definitions

The Padua Prediction Score assigns **3 points** for “reduced mobility ≥3 days,” but the original definition is vague:

“Bed rest with bathroom privileges (apart from bathroom needs) due to patient limitations or on physician’s order for at least 3 days.”

## Key Problems:

1. **“Bathroom privileges”** is ambiguous (walking 5 meters? wheelchair transfer?)
2. **“Patient limitations”** versus **“physician’s order”** creates different assessment contexts
3. **No validated assessment tool** exists for retrospective chart review
4. **Inter-rater reliability unknown** in real-world settings

# Proposed Templates for Prospective Validation

**Five templates** spanning quantitative, functional, objective, structured, and hybrid approaches. Each should be pilot-tested in a nested substudy to determine which best predicts pulmonary embolism (PE) risk.

## TEMPLATE 1: Percentage-Based Mobility Reduction (Quantitative)

**Assessment Question:** *“Compared to the patient’s baseline mobility 2 weeks ago, their current mobility is reduced by:”*

| Category | Definition | Padua Points |
| --- | --- | --- |
| 0% | Unchanged from baseline | 0 |
| 1%-24% | Slightly reduced (can walk but less than usual) | 0 |
| 25%-49% | Moderately reduced (mostly walking short distances) | **3** |
| 50%-74% | Severely reduced (mostly chair-bound, walks <10 meters/day) | **3** |
| 75%-100% | Bedbound (cannot walk, transfers only to chair/toilet) | **3** |

**Advantages:**

- Intuitive for clinicians
- Captures graded severity

**Disadvantages:**

- Requires baseline knowledge (may be unavailable in acute settings)
- Subjective percentage estimation

**Validation Method:** Compare against actigraphy (step count) in subsample

## TEMPLATE 2: Functional Activity Scale (3-Day Recall)

**Assessment Question:** *“Over the past 3 days, the patient has been:”*

| Level | Description | Examples | Padua Points |
| --- | --- | --- | --- |
| Level 0 | Normal activity | Walking >500 m/day, shopping, household tasks | 0 |
| Level 1 | Limited activity | Walking 100-500 m/day, sitting most of day but mobile | 0 |
| Level 2 | Chair-dependent | Sitting >20 hours/day, walking only to bathroom (<50 m/day) | **3** |
| Level 3 | Bed-dependent | Lying >20 hours/day, transfers only with assistance | **3** |
| Level 4 | Fully bedbound | Cannot transfer without mechanical lift | **3** |

**Advantages:**

- Clear behavioral anchors
- Aligns with WHO Performance Status concepts
- Easy to document in nursing notes

**Disadvantages:**

- Requires reliable patient/caregiver recall

**Validation Method:** Concordance with nurse-reported mobility documentation

## TEMPLATE 3: Objective Measurement (Wearable Device)

**Assessment Method:** *Step count measured via wearable accelerometer (Fitbit, Apple Watch, hospital-issued device) over 72 hours*

| Step Count (3-day average) | Interpretation | Padua Points |
| --- | --- | --- |
| >3,000 steps/day | Adequate mobility | 0 |
| 1,000-3,000 steps/day | Reduced but not severely | 0 |
| <1,000 steps/day | Severely reduced mobility | **3** |

**Advantages:**

- Objective, reproducible
- No recall bias

**Disadvantages:**

- Requires technology infrastructure
- May not capture bed-to-chair transfers (underestimates immobility)
- Unsuitable for intensive care unit (ICU) patients

**Validation Method:** Gold standard for Template 1 and 2 comparisons

## TEMPLATE 4: Structured Clinical Observation (Nurse-Rated)

**Assessment Tool:** Adapted from Johns Hopkins Highest Level of Mobility (JH-HLM) Scale

*Nurse rates the highest level of mobility achieved in past 3 days:*

| Mobility Level | Description | Padua Points |
| --- | --- | --- |
| **1** | Walking without assistance (>50 meters) | 0 |
| **2** | Walking with/without assistance (10-50 meters) | 0 |
| **3** | Transfer to chair, no walking | **3** |
| **4** | Sitting at edge of bed, no standing | **3** |
| **5** | Lying in bed, passive range of motion only | **3** |

**Advantages:**

- Already used in hospital quality metrics
- High inter-rater reliability (validated in ICU/ward settings)
- Real-time documentation

**Disadvantages:**

- Requires nursing staff training

**Validation Method:** Correlation with 90-day venous thromboembolism (VTE) incidence

## TEMPLATE 5: Hybrid Template (Clinical Judgment + Objective Trigger)

**Two-Step Assessment:**

**STEP 1 (Screening Question):** *“Has the patient been out of bed for <1 hour total per day (excluding bathroom) for ≥3 days?”*

- **YES** → Proceed to Step 2
- **NO** → Assign 0 points

**STEP 2 (Objective Confirmation):** *Document one of the following:*

- Physician order for bed rest
- Nursing note: “Patient bedbound” or “Chair-bound, no ambulation”
- Physical therapy note: “Unable to ambulate”
- Step count <500/day (if available)

If any criterion met → **Assign 3 points**

**Advantages:**

- Combines subjective and objective data
- Minimizes false positives (screening question filters out borderline cases)

**Disadvantages:**

- Two-step process may reduce compliance

# Recommended Pilot Study Design

## Phase 1: Template Comparison Study (*n*=200 patients)

**Objective:** Determine which template best predicts PE risk

**Methods:**

1. Enroll 200 consecutive patients aged ≥65 with suspected PE
2. Apply **all 5 templates** to each patient (blinded assessors)
3. Record:
   - Final PE diagnosis (reference standard)
   - Inter-rater reliability (2 independent assessors per template)
   - Time to complete each assessment
4. Primary outcome: **Area under ROC curve** for PE prediction
5. Secondary outcomes:
   - Inter-rater reliability (Cohen’s kappa)
   - Feasibility (% missing data)
   - Clinician preference survey

## Phase 2: Prospective PaDd Validation (*n*=1,000 patients)

**Objective:** Validate PaDd using best-performing template from Phase 1

**Methods:**

1. Use the selected template to calculate Padua score
2. Compute PaDd (Padua × D-dimer)
3. Compare diagnostic accuracy versus age-adjusted D-dimer alone
4. Report outcomes stratified by mobility template threshold

## Anticipated Results by Template

| Template | Predicted Strength | Predicted Weakness | Best Use Case |
| --- | --- | --- | --- |
| **Template 1** | Captures gradations | Subjective estimation | Retrospective studies |
| **Template 2** | Clear definitions | Recall bias | Prospective cohorts |
| **Template 3** | Objective | Technology barrier | Research hospitals |
| **Template 4** | Already validated | Requires training | Pragmatic trials |
| **Template 5** | Balances rigor/feasibility | Two-step complexity | Clinical implementation |

**Recommendation:**

**Templates 2, 4, and 5** are identified as the most feasible options for immediate prospective validation, while **Template 3** may serve as an optional objective comparator in well-resourced sites where additional analytical rigor or benchmarking against established approaches is desired.
